# Meclizine moderates lipopolysaccharide-induced neuroinflammation in mice through the regulation of AKT/ NF-κβ/ERK/JNK signaling pathway

**DOI:** 10.1007/s11011-023-01295-3

**Published:** 2023-09-21

**Authors:** Rasha E. Mostafa, Gihan F. Asaad

**Affiliations:** Department of Pharmacology, Medical Research and Clinical Studies Institute, National Research Centre, 33 ELBohouth St. (former EL Tahrir St.), P.O. 12622, Dokki, Cairo Egypt

**Keywords:** Meclizine, Lipopolysaccharide, Neuroinflammation, NF-κB, ERK, JNK

## Abstract

Neuroinflammation is identified as significant inflammatory reactions occurring in the central nervous system. Lipopolysaccharide (LPS) stimulates innate immune reactions and is used as an in vivo animal model for the investigation of inflammation. Meclizine (MCLZ) is a histamine antagonist with potential neuroprotective qualities. Forty adult male Swiss albino mice were divided into four groups (n = 10). Group 1 served as a control negative group. Groups 2–4 were injected with LPS (5 mg/kg; i.p). Group 2 served as LPS-control. Groups 3 & 4 were given MCLZ (12.5 & 25 mg/kg; p.o) respectively for 14 days. LPS administration resulted in significant neuroinflammation in mice as was revealed by significant inflammatory histopathological changes and positive immunohistochemical staining of glial fibrillary acidic proteins (GFAP) accompanied by significant elevations of brain tissue contents of interleukin-1-beta (IL-1β), tumor necrosis factor-alpha (TNF-α), nuclear factor kappa-beta (NF-κβ), protein kinase B (AKT), extracellular signal-regulated kinase (ERK) and C-Jun N-Terminal Kinases (JNK). MCLZ treatment significantly down-regulated all the aforementioned parameters in mice brains. Moreover, MCLZ treatment ameliorated the inflammatory histopathological changes and GFAP immunostaining in brain tissues. The current study identifies for the first time the protective anti-neuroinflammatory effects of MCLZ against LPS-induced neuroinflammation in mice. MCLZ protected against neuroinflammation via the amelioration of inflammatory histopathological changes as well as neuronal GFAP immunostaining and down-regulated the AKT/NF-κβ/ERK/JNK signaling pathway. MCLZ is anticipated as a potential protective candidate for the addition to the treatment protocol of neuroinflammation.

## Introduction

Neuroinflammation is usually identified as a significant inflammatory reaction occurring in the central nervous system (CNS). These inflammatory reactions are triggered by cytokines, chemokines and inflammatory enzyme release. The CNS, which is a structure isolated and protected by the blood-brain barrier, has localized immune cells, viz., microglia and astrocytes. (Skrzypczak-Wiercioch and Sałat 2022). Microglial cells are a special kind of glial cells linked to macrophages. They comprise the main immune cells in the brain & spinal cord. They are commonly referred to as “housekeeping cells” since they are crucial for maintaining homeostasis, eliminating metabolic waste products, and cell debris as well as responding to neuroinflammation. This can be attributed to their capacity to take on various morphologies and can play double roles in neuronal injury and recovery (Gargouri et al. 2018). On the other hand, astrocytes provide a variety of purposes where they play a crucial function in maintaining BBB and are fundamental for both the growing and adult brain. Neuroinflammation accompanied by excessive microglia as well as astrocyte activation is the initial indicator of most neurological disorders (Jiang et al. 2022).

To date, there is no effective treatment for neuroinflammatory and neurodegenerative diseases. In general, little is currently known about the mechanisms underlying the onset and progression of such diseases. Neuroinflammation is seen as a major contributor to neurodegenerative diseases; among other variables. Significant experimental evidence has shown that neuronal cell death can cause inflammation, and conversely, inflammation on its own can cause neuronal cell death (Batista et al. 2019).

Lipopolysaccharide (LPS), the primary component of Gram-negative bacteria’s outer cell wall, has been utilized as a traditional stimulant to elicit innate immune reactions. LPS has been widely used as an in vivo animal model for the investigation of peripherally- and centrally-induced inflammation (Wu et al. 2015). On the molecular level, LPS initiates a sequence of changes in protein expression where NF-κβ nuclear translocation is activated leading to further production of pro-inflammatory cytokines (Li et al. 2020). Via the activation of vagal afferent neurons, intraperitoneal injections of LPS cause the transfer of peripheral inflammation to the brain (Pavlov and Tracey 2012). Moreover, peripheral pro-inflammatory cytokines have the ability to cross the BBB and activate NF-κβ by binding to their specific receptors in the brain endothelium. LPS worsens learning and memory in mice and raises levels of plasma, hippocampal, and cortical pro-inflammatory cytokines (Cazareth et al. 2014). As a result, astrocytes and microglia become abnormally activated and consequently release pro-inflammatory proteins, which heighten the neuroinflammatory response to LPS administration (Ryu et al. 2019).

Meclizine is a piperazine-derived histamine antagonist. It has been used for many years as an “over-the-counter” H1 receptor blocker to alleviate nausea, vomiting, and dizziness brought on by motion sickness. It is a commonly used, well-tolerated medication to control disequilibrium. Lately, meclizine has been investigated for its neuroprotective qualities in many neurological disorders such as ischemic stroke, Parkinson’s disease and Huntington’s disease (Hong et al. 2016).

Henceforth, the current study aims to scout for the potential anti-neuroinflammatory effects of meclizine against LPS-induced neuroinflammation in mice. This study also investigates the possible molecular signaling pathways underlying these possible anti-neuroinflammatory actions.

## Materials and methods

### Animals

Forty adult male Swiss albino mice weighing (25–30 g) were purchased from the animal breeding unit at the National Research Centre. Animal housing guidelines have been created, including a 12:12 light-dark cycle and well-ventilated chambers. The animals were housed in hygienic plastic cages and provided with a clean standard pellet diet meal as well as unlimited access to water. The study was conducted following the recommendations of the Helsinki Declaration, and good medical & laboratory practices (GCP and GLP). National Research Centre–Medical Research Ethics Committee (NRC-MREC) for the use of animal subjects granted ethical approval for the current study (Approval number 1,414,052,023).

### Drugs and chemicals

Lipopolysaccharide (LPS) was purchased from Sigma Aldrich, Germany. Meclizine (MCLZ) was purchased as meclizine hydrochloride 25 mg oral dissolvable films (Nerhadou International for Pharmaceuticals and Nutraceuticals, Giza, Egypt). ELISA kits were purchased as follows: Interleukin-1-beta (IL-1β; Cloud Clone Corp., TX77494, USA), tumor necrosis factor-alpha (TNF-α; BioLegend, Inc., CA 92,121, USA), nuclear factor kappa-beta (NF-κβ; CUSABIO, TX 77,054, USA), protein kinase B (AKT; MyBioSource, CA 92,195 − 3308, USA), extracellular signal-regulated kinase (ERK; MyBioSource, CA 92,195 − 3308, USA) and C-Jun N-Terminal Kinases (JNK; Wuhan, China). All ELISA tests were conducted according to the manufacturer’s instructions and all chemicals used were of the highest commercial grade. The absorbance was read for all parameters at 450 nm using an ELISA plate reader (Stat Fax 2200, Awareness Technologies, Florida, USA).

### Experimental design and treatment protocol

**Fig. 1:**
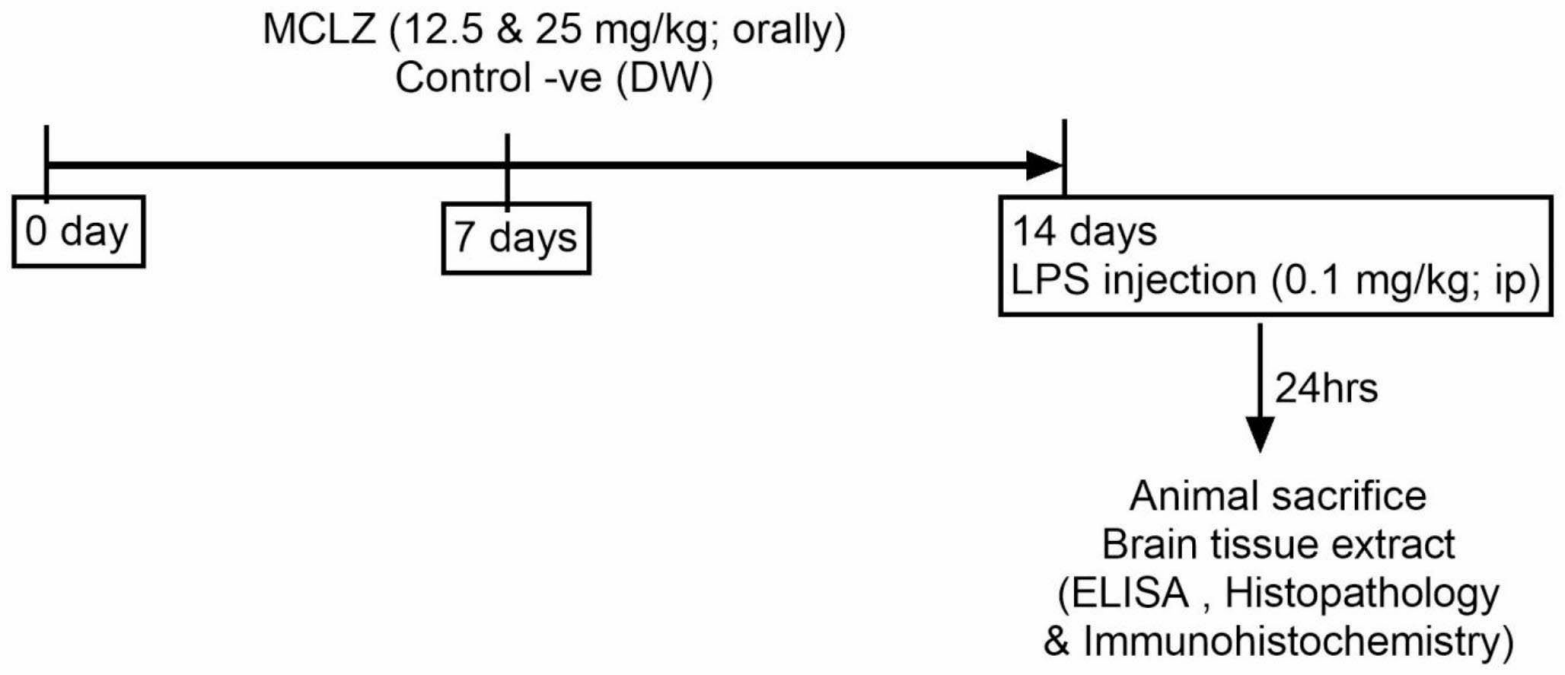
Schematic diagram of experimental design for LPS-induced neuroinflammation and prophylactic administration of MCLZ (12.5&25 mg/kg, p.o)

Forty adult male Swiss albino mice weighing (25–30 g) were randomly allocated into four groups (n = 10). Group 1 was administered distilled water (DW) orally for 14 days and served as a control negative group. Group 2 was injected with a single dose of LPS (5 mg/kg) intraperitoneally (Batista et al. 2019) after oral administration of DW for 14 days and served as LPS-control. Groups 3 and 4 were given oral meclizine (MCLZ; 12.5 & 25 mg/kg) (Singh et al. 2020) respectively for 14 days and at day 14, mice were injected intraperitoneally with LPS (5 mg/kg). Twenty-four hours later, all mice were sacrificed and brains were extracted. One portion of the brain was kept in -80°C till analysis and another portion was kept in 10% formalin for histopathological examination (**Fig. **1).

### Brain tissue homogenate for ELISA assessment

Brain tissues were homogenized (MPW-120 homogenizer, Med instruments, Poland) in phosphate-buffered saline to obtain 20% homogenate. The homogenates were centrifuged for 15 min at 5000 x g using a cooling centrifuge (Sigma and laborzentrifugen, 2k15, Germany). All results are calculated per 1 mg of total protein.

### Histopathological examination of brain tissues

Brains from all experimental groups were fixed in 10% neutral buffered formalin. The fixed samples were dehydrated in ascending series of ethanol, cleared in xylene, and embedded in paraffin wax. Section 5 μm thickness were prepared using a microtome, stained with hematoxylin and eosin (H & E), and examined under a light microscope.

### Immunohistochemical staining of glial fibrillary acidic proteins (GFAP)

Paraffin sections were mounted on positively charged slides by using avidinbiotin- peroxidase complex (ABC) method. Mouse Anti GFAP monoclonal antibodies (Servicebio, Cat # GB12100, Dil 1:800) are used in the current work. Sections from each group are incubated with these antibodies, and then the reagents required for the ABC method are added (Vectastain ABC-HRP kit, Vector laboratories). Marker expression is labeled with peroxidase and colored with diaminobenzidine (DAB, produced by Sigma) to detect antigen-antibody complex. Negative controls are included using non-immune serum instead of the primary or secondary antibodies. IHC stained sections were examined via an Olympus microscope (BX-53).

Scoring of immunohistochemical results is performed via the determination of the reaction area percent in 10 microscopic fields using image J 1.53t software(Wayne Rasband and contributors, National Institutes of Health, USA) (Mostafa et al. 2021a).

### Statistical analysis

All the values are presented as means ± standard error of the means (SEM). Statistical analysis was performed using one-way ANOVA and followed by *Tukey-Kramer* test for confirmation. p˂0.05 was considered significant. GraphPad prism® Software, Inc.(San Diego, CA, USA) was used to carry out all statistical tests.

## Results

### Effects of meclizine on IL-1β-TNF-α-NF-κβ signaling in LPS-induced neuroinflammation in mice

In the current study, a single administration of LPS (5 mg/kg; IP) significantly (****p < 0.0001) elevated IL-1β, TNF-α and NF-κβ (198.1 ± 6.38, 137 ± 1.67 and 30.82 ± 0.98 pg/mg protein) respectively, showing 5.6, 4.95, and 6.5 fold increase respectively as compared to the control levels of IL-1β (35.44 ± 0.78 pg/mg protein), TNF-α (27.65 ± 1.45 pg/mg protein) and NF-κβ (4.75 ± 0.13 pg/mg protein); respectively.

Administration of MCLZ at both dose levels (12.5 & 25 mg/kg; p.o) for 14 days before induction of neuroinflammation via LPS resulted in a marked reduction in the induced inflammatory cytokines, whereas the administration of MCLZ (12.5 mg/kg; p.o) for 14 days showed a significant (****p < 0.0001) reduction in IL-1β (141.3 ± 4.37 pg/mg protein; 1.4 fold), TNF-α (100.2 ± 0.78 pg/mg protein; 1.37 fold) and NF-κβ (19.29 ± 0.76 pg/mg protein; 1.59 fold) as compared to the LPS-control group; respectively. Also, the administration of MCLZ at the higher dose (25 mg/kg) significantly (****p < 0.0001) inhibited the induced inflammatory biomarkers; IL-1β (51.75 ± 0.92 pg/mg protein; 3.82 fold), TNF-α (45.51 ± 0.68 pg/mg protein; 3.01 fold) and NF-κβ (7.59 ± 0.23 pg/mg protein; 4.06 fold) as compared to the LPS-control group.

**Fig. 2:**
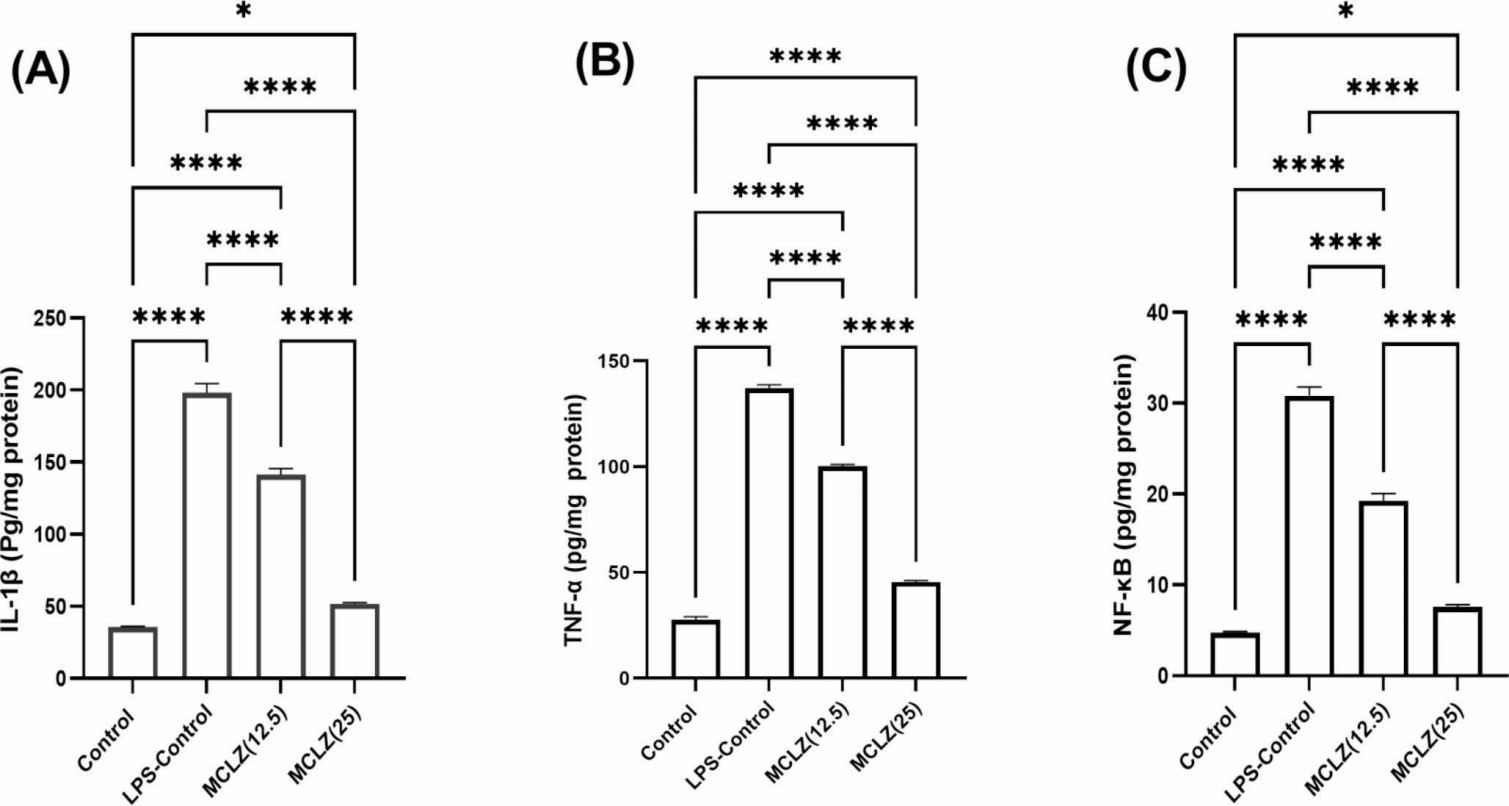
Effects of Meclizine on IL-1β-TNF-α-NF-κβ signaling in LPS-induced neuroinflammation in mice Data is presented as mean ± SEM (n = 10). Statistical analysis was conducted by one-way analysis of variance (ANOVA) followed by *Tukey-Kramer’s* test for multiple comparisons (*p < 0.05, ****p < 0.0001) MCLZ; Meclizine, LPS; Lipopolysaccharide, IL-1β; Interleukin − 1 beta, TNF-α; Tumor necrosis factor-alpha, NF-κβ; Nuclear factor kappa- beta

Accordingly, we recorded that the high dose of MCLZ (25 mg/kg) significantly (****p < 0.0001) inhibited the LPS-induced IL-1β, TNF-α and NF-κβ by a range of ≈ 2-2.7 folds as compared to the lower dose (12.5 mg/kg). Here, we demonstrated that the triggered NF- κβ signaling pathway was brought on by the induction of proinflammatory cytokines; IL-1β, and TNF-α via LPS injection and as per our results, we can conclude that the administration of MCLZ can interestingly ameliorate the IL-1β-TNF-α-NF-κβ signaling pathway; dose-dependently, and endorse the anti-inflammatory impact of meclizine against neuroinflammation induced by LPS. These results are depicted in **Fig. **2.

### Effects of meclizine on AKT-ERK-JNK signaling in LPS-induced neuroinflammation in mice

In the current work, a single administration of LPS (5 mg/kg; IP) significantly (****p < 0.0001) elevated AKT, ERK and JNK (29.65 ± 1.72, 10.74 ± 0.63 and 5.42 ± 0.08 ng/mg protein) respectively, showing 4.26, 4.71, and 4.75 fold increase respectively as compared to the control levels of AKT (6.95 ± 0.13 ng/mg protein), ERK (2.28 ± 0.07 ng/mg protein) and JNK (1.14 ± 0.08 ng/mg protein).

**Fig. 3:**
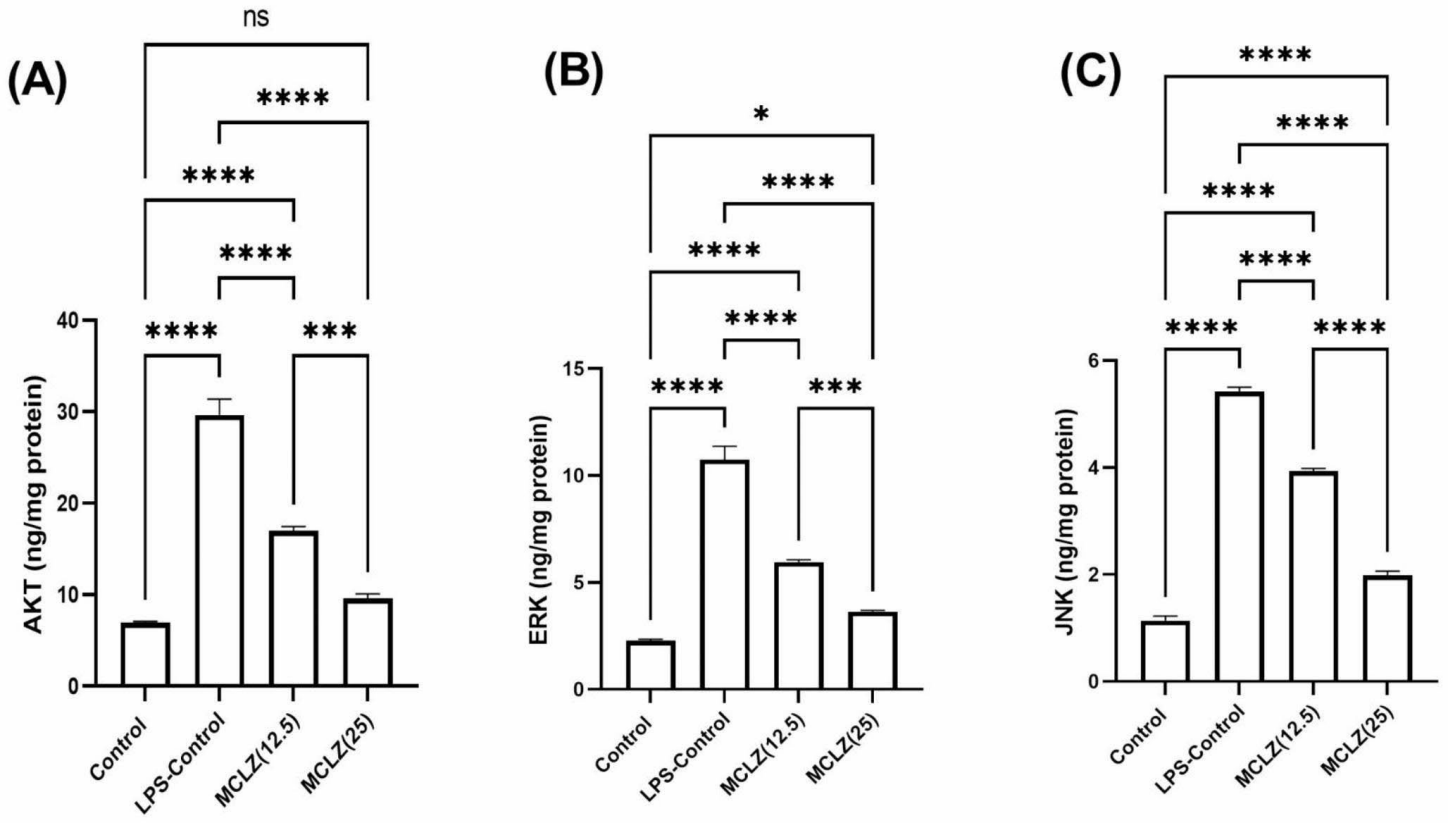
Effects of Meclizine on AKT-ERK-JNK signaling in LPS-induced neuroinflammation in mice Data is presented as mean ± SEM (n = 10). Data is analyzed by one-way ANOVA followed by Tukey’s post hoc test, Statistical analysis was conducted by one-way analysis of variance (ANOVA) followed by *Tukey-Kramer’s* test for multiple comparisons (*p < 0.05, ***p < 0.001, ****p < 0.0001) MCLZ; Meclizine, LPS; Lipopolysaccharide, AKT; Protein kinase B, ERK; Extracellular signal-regulated kinase, JNK; c-Jun N-terminal Kinase

MCLZ administration (12.5 mg/kg; p.o) for 14 days showed a significant (****p < 0.0001) reduction in AKT (16.97 ± 0.48 ng/mg protein; 1.74 fold), ERK (5.96 ± 0.09 ng/mg protein; 1.8 fold) and JNK (3.93 ± 0.05 ng/mg protein; 1.38 fold) as compared to the LPS-control group. Additionally, the administration of MCLZ at the higher dose (25 mg/kg, p.o) significantly (****p < 0.0001) inhibited the induced inflammatory biomarkers; AKT (9.626 ± 0.46 ng/mg protein; 3.08 fold), ERK (3.63 ± 0.07 ng/mg protein; 2.95 fold) and JNK (1.98 ± 0.08 ng/mg protein; 2.74 fold) as compared to LPS-control group. So, we demonstrated that the high dose of MCLZ (25 mg/kg) significantly inhibited the LPS-induced AKT (***p < 0.001), ERK (***p < 0.001) and JNK (****p < 0.0001) by a range of ≈ 1.6-2 folds as compared to the lower dose (12.5 mg/kg). We may conclude that meclizine administration can interestingly improve the AKT-ERK-JNK signaling pathway; dose-dependently, and support the anti-inflammatory effects of the drug against neuroinflammation brought on by LPS. These results are depicted in Fig. 3.

### Histopathological examination of brain tissues

**Fig. 4:**
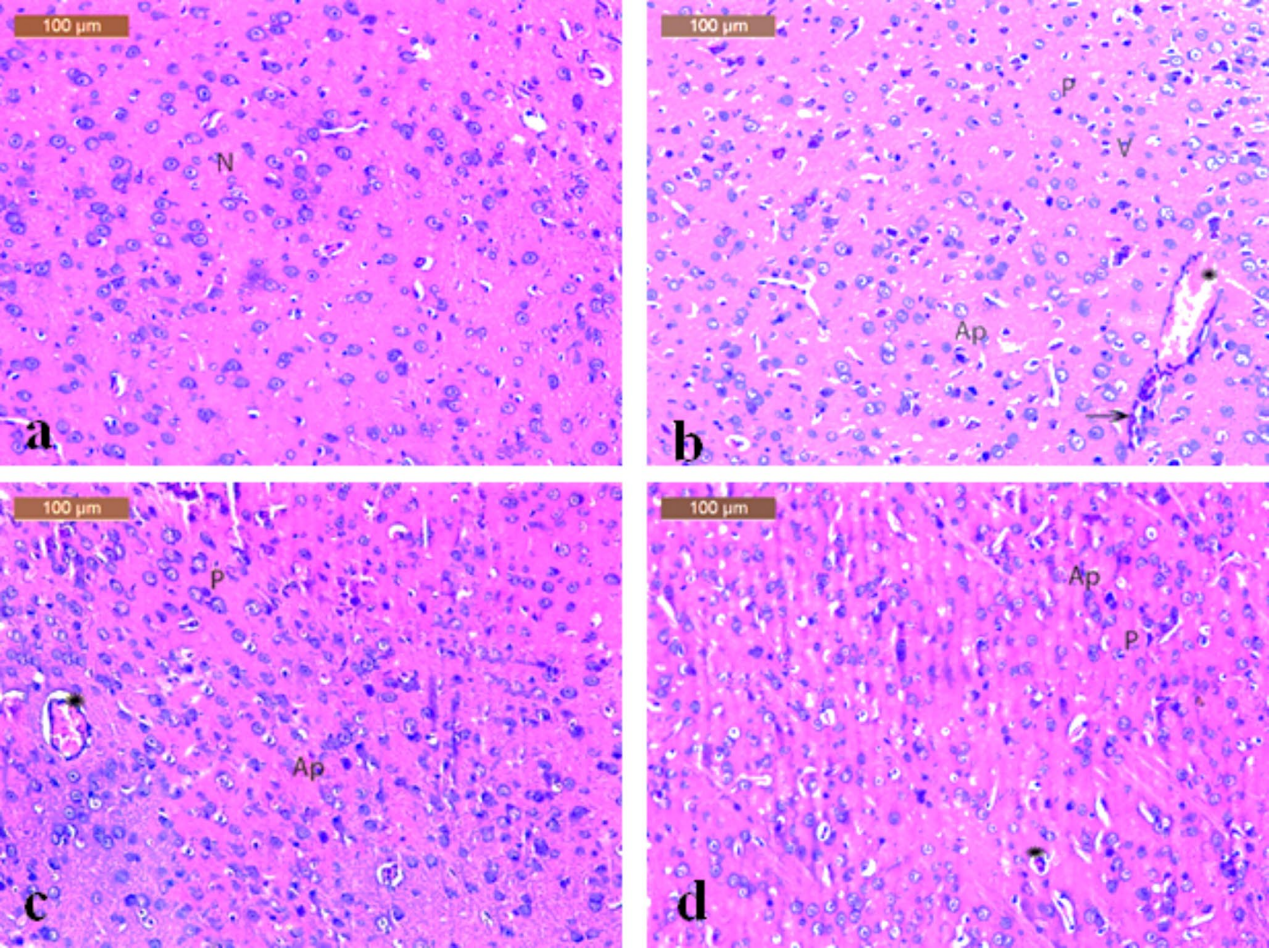
Histopathological examination of brain tissue Photomicrographs of sections of (**a**) a brain of the control group showing a normal histological structure of cortex with normal neurons (N), (**b**) a brain of the LPS-control group showing degeneration of neuronal architecture along with vacuolated cells (V), apoptotic cells (Ap), dark pyknotic nuclei (P), inflammatory cells (arrow) with dilated congested blood vessel (Star), (**c**) a brain of the group treated LPS + MCLZ (12.5 mg/kg, p.o) showing moderate ameliorative effects with mild apoptotic cells (Ap), mild pyknotic nuclei (P), with congested blood vessel (Star), and (**d**) a brain of the group treated with LPS + MCLZ (25 mg/kg, p.o) showing noticeable ameliorative effect with few apoptotic cells (Ap), mild pyknotic nuclei (P), with blood vessel (Star). (Stain: H&E; scale bar = 100 μm)

The brain sections from the control group showed normal architecture of the cortex with neurons being arranged in neat rows with abundant cytoplasm, prominent nucleoli and vesicular nuclei (**Fig. **4a). Histopathological changes of LPS-control group were apparent as degeneration of neuronal architecture. Moreover, vacuolated cells, apoptotic cells, dark pyknotic nuclei, and inflammatory cells with dilated congested blood vessels were also noticed (**Fig. **4b). Sections from the group treated with LPS and oral meclizine (12.5 mg/kg) showed moderate ameliorative effects and less histopathological changes with mild apoptotic cells, mild pyknotic nuclei and congested blood vessel (**Fig. **4c).

(25 mg/kg) showed noticeable ameliorative effects whereas few apoptotic cells, mild pyknotic nuclei, with blood vessels could be observed (**Fig. **4d).

### Immunohistochemical staining of glial fibrillary acidic proteins (GFAP)

**Fig. 5:**
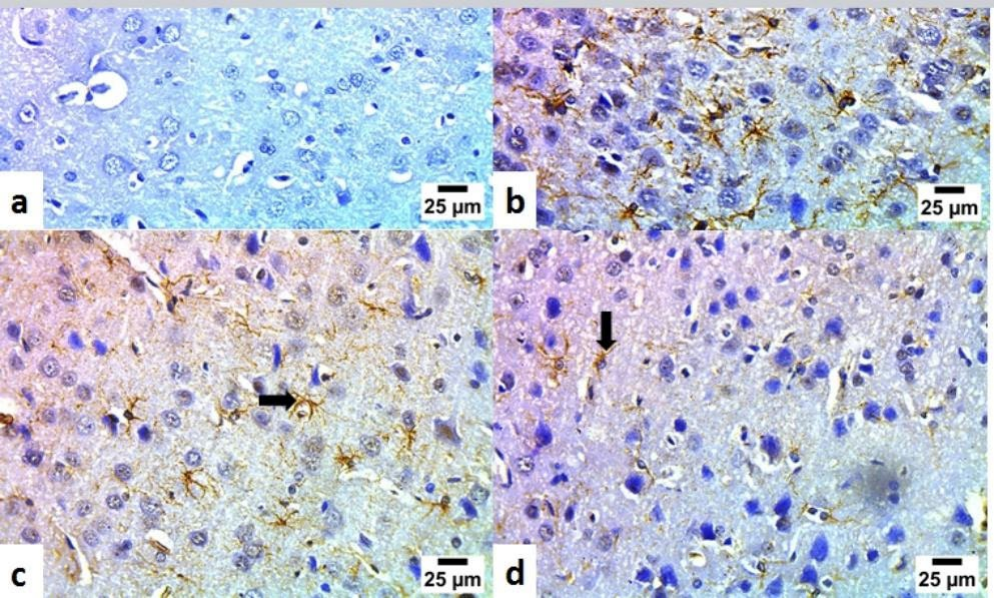
Immunohistochemical staining of glial fibrillary acidic proteins (GFAP) in brain tissue Photomicrographs of sections of (**a**) a brain of the control group showing a normal negative neuronal expression of GFAP (arrow), (**b**) a brain of the LPS-control group showing severe positive neuronal expression of GFAP (arrow), (**c**) a brain of the group treated LPS + MCLZ (12.5 mg/kg, p.o) showing moderate positive neuronal expression of GFAP (arrow), and (**d**) a brain of the group treated with LPS + MCLZ (25 mg/kg, p.o) showing very mild positive neuronal expression of GFAP (arrow). (**GFAP immunostaining; scale bar = 25 μm**)

The brain sections from the control group showed negative neuronal expression of GFAP (**Fig. **5a). Immunohistochemical staining of the LPS-control group showed severe positive neuronal expression of GFAP (**Fig. **5b). Immunohistochemical staining of sections from the group treated with LPS and oral meclizine (12.5 mg/kg) showed moderate positive neuronal expression of GFAP (**Fig. **5c). On the other hand, immunohistochemical staining of sections from the group treated with LPS and oral meclizine (25 mg/kg) showed very mild positive neuronal expression of GFAP (**Fig. **5d). The scoring of GFAP immunohistochemical expression in neurons of normal and treated mice is illustrated in Table 1.

**Table 1:** Scoring of the GFAP immunohistochemical expression in neurons of normal and treated mice

Groups	GFAP immunohistochemical expression (Reaction area % in 10 microscopic fields)
Control	0.3^a^ ± 0.06
LPS-Control	10.5^b^ ± 0.54
**MCLZ (12.5 mg/kg**)	**4.8**^**c**^ **± 0.27**
**MCLZ (25 mg/kg**)	**1.8**^**d**^ **± 0.12**

Different lowercase letters are significantly different (p < 0.05). Image J 1.53t software was used for the determination of the reaction area percent. GFAP: glial fibrillary acidic proteins

## Discussion

Huge evidence indicates that high levels of pro-inflammatory cytokines encourage neuronal malfunction and death, which is why neuroinflammation has been linked to the emergence of neurodegenerative diseases. Therefore, it is crucial to research potential substances that reduce the inflammatory response in the central nervous system. The current study is the first to evaluate the prophylactic anti-neuroinflammatory effects of meclizine against LPS-induced neuroinflammation in mice. This study is also extended to look out for the possible molecular signaling pathways mediating these possible anti-neuroinflammatory actions.

In the current work, the intraperitoneal injection of LPS (5 mg/kg) caused significant neuroinflammation in mice as was revealed by significant elevation of brain tissue contents of IL-1β, TNF-α, NF-κβ, AKT, ERK and JNK. Moreover, significant inflammatory histopathological changes along with positive GFAP immunohistochemical staining have been noticed in mice brains following LPS administration. Meclizine oral prophylactic administration resulted in significant anti-inflammatory effects.

LPS -an endotoxin isolated from the Gram-negative bacterial cell wall- is capable of inducing neuroinflammation, and neurotoxicity along with cognitive impairment in experimental animals. Numerous signaling pathways have been proposed to elucidate LPS-induced neuroinflammation, however, the exact mechanisms have not yet been well-identified (Fasolo et al. 2021). Neuroinflammation is a cascade of inflammatory reactions that occur in the CNS, accompanied by the massive production of cytokines, chemokines and inflammatory enzymes. The CNS contains two main types of immunocompetent cells, viz., microglia and astrocytes. Microglia maintain homeostasis in the CNS where they sense and remove excessive metabolic waste and cellular debris. Likewise, astrocytes play a crucial function in maintaining BBB and are fundamental for both the growing and adult brain. Therefore, both microglia and astrocytes play an essential role in neuroinflammation (Skrzypczak-Wiercioch and Sałat 2022).

LPS administration resulted in significant activation of the microglia. Microglial activation stimulates the triggering of pro-inflammatory mediators such as NF-kβ, TNF-α, IL-16, IL-1β, NO, and reactive oxygen species (ROS) to stimulate tissue repair (Gargouri et al. 2018). It is well established that the NF-κβ pathway is essential for the CNS, especially in cases of brain inflammation, acute cerebral traumas, and neurodegenerative diseases (Mostafa et al. 2021a). NF-κβ is crucial for the microglia-mediated inflammatory reaction in the CNS (Raasch et al. 2011). NF-kβ signaling initiates inflammatory responses via the activation of numerous pro-inflammatory cytokines in most cells (Mostafa et al. 2022). Cytokines are small polypeptides produced by a variety of cells controlling cell growth, differentiation, inflammation and wound healing (Asaad and Mostafa 2022). NF-kβ signaling causes the activation of TNF-α and interleukins. Dysregulation in NF-kβ signaling justifies the pathogenesis of numerous inflammatory syndromes (Mostafa et al. 2021b).

Similar to our work, many studies suggested the involvement of NF-kβ signaling in LPS-induced neuroinflammation (Batista et al. 2019; Guo et al. 2022; Skrzypczak-Wiercioch and Sałat 2022; Yegla and Foster 2019). Gargouri et al. (2018) reported stimulation of TNF-α, IL-1β along with IL-6 in LPS-activated primary microglial cells (Gargouri et al. 2018). Another study reported TNF-α and IL-1β release from both astrocytes and microglia in response to neuroinflammation following LPS administration in mice (Rojas-Colón et al. 2021).

Numerous inflammatory diseases, malignancies and autoimmune conditions stimulate the AKT signaling pathway. It is well-documented that effective NF-kβ signaling and the expression of NF-kβ-regulated inflammatory cascade depend on the AKT activation (Mostafa and Salama 2023). NF-κβ is the main downstream target for AKT (Mostafa and Abdel-Rahman 2023).

Similar to the current work, reported stimulation of AKT by LPS-induced neuroinflammation in LPS-activated microglia (Zhao et al. 2019). A key regulator of IL-6 and TNF-α is ERK. It is noteworthy that ERK production is excessively triggered in several inflammatory and auto-immune disorders (Mostafa and Salama 2023). JNKs are members of the family of mitogen-activated protein kinases that are activated by a variety of stimuli, such as inflammation, oxidative stress, and brain ischemia-reperfusion injury. JNKs are expressed in all cells and tissues throughout the body. JNKs are thought to play a crucial role in the processes involved in neuronal injury and are usually involved in the development of neuroinflammation, stroke, Alzheimer’s disease and Parkinson’s disease. Therefore, JNK inhibitors have potential neuroprotective effects (Anfinogenova et al. 2020). Similar to the current work, Lim et al. (2018) suggested that LPS-induced neuroinflammatory changes in mice brains may be attributed to NF-κB along with several mitogen-activated protein kinases; viz., ERK and JNK (Lim et al. 2018). Consistent data was also reported where mitogen-activated protein kinases signaling, including ERK, and JNK up-regulation initiate numerous neuroinflammatory mediators (Jung et al. 2016; Qi et al. 2016). Similarly, Guo et al. (2022) reported the involvement of the ERK/JNK/NF-κB signaling pathway in LPS-induced neuroinflammation induced experimentally in mice (Guo et al. 2022).

GFAP is an immunohistochemical indicator overexpressed in response to neuronal and astrocyte inflammatory injury. In the context of neuroinflammation, GFAP expression in astrocytes is the key element of the glial scar formation (Mostafa et al. 2021a). The current study reveals a significant elevation in the GFAP immunohistochemical reactivity expressed as reaction area percent in LPS-control mice. In line with this finding, many studies reported that LPS causes abundant cellular proliferation neuroinflammation and GFAP expression in experimental injuries of the CNS in rodents and human cell lines. (Gao et al. 2020; Niranjan et al. 2014).

Meclizine, a histamine antagonist derived from piperazine, is currently used as an “over-the-counter” H1 receptor blocker to reduce motion sickness-related nausea, vomiting, and dizziness. It is a widely prescribed, well-tolerated drug. Meclizine’s neuroprotective properties in a variety of neurological conditions, including ischemic stroke, Parkinson’s disease and Huntington’s disease, have recently come under investigation (Hong et al. 2016).

Meclizine seems to be usually well tolerated overall. Among its most frequent side effects is sleepiness. Numerous investigations concluded that meclizine had little to no effect on perceptual efficiency and detrimental memory (Patel and Ambizas 2011). Studies examining the CNS effects of meclizine 50 mg found that it significantly increased recognition and reaction time (Manning et al. 1992). The US Food and Drug Administration (FDA) declared that there is insufficient evidence to justify restricting the use of meclizine during pregnancy. (Patel and Ambizas 2011). Preclinical safety studies of repeated oral administration of meclizine once or twice a day for 14 days appeared to be safe and well-tolerated with no serious adverse events (Kitoh et al. 2020).

MCLZ use in the current work was linked to significant anti-inflammatory effects as manifested by a statistically significant reduction in brain tissue concentration of brain tissue contents of IL-1β, TNF-α, NF-κβ, AKT, ERK and JNK. Additionally, significant amelioration of the histopathological inflammatory changes and GFAP immunohistopathological staining have been noticed in the brains of mice treated with MCLZ.

MCLZ has been previously reported to cross the blood-brain barrier and to possess cytoprotective and anti-neurodegenerative actions. These effects are independent of its anti-muscarinic or anti-histaminergic actions (Gohil et al. 2011).

Earlier studies reported that MCLZ’s neuroprotective effects comprise suppression of apoptosis along with increased glycolysis without affecting levels of total ATP (Hong et al. 2016).

Singh et al. (2020) stated that MCLZ spares cognitive functions in mice due to its antioxidant and anti-inflammatory actions. MCLZ administration caused a significant reduction in proinflammatory cytokines (IL-1β & TNF-α) in mice brains post streptozotocin administration (Singh et al. 2020). Another study reported that MCLZ decreased mitochondrial oxygen consumption, oxidative stress and inflammation as manifested by reduced IL-6, IL-1β & TNF-α in kidney tissues of mice (Kishi et al. 2015). Guo et al. (2017) reported that MCLZ attenuated ovariectomy-induced bone loss via attenuation of NF-κβ, ERK and p38 (Guo et al. 2017). A recent study also highlighted the possible anti-inflammatory effects of MCLZ where it inhibits the activation of the JNK and NF-κβ and inflammatory cytokines in allergic airway inflammation in mice (Jang et al. 2023).

To the authors’ knowledge, the current work is the first to assess MCLZ’s protective anti-neuroinflammatory effects. The study suggests that MCLZ’s effects are in virtue of the amelioration of the AKT/NF-κβ/ERK/JNK signaling pathway. Based on all the collected results, MCLZ can be of value in protecting against neuroinflammatory disorders. Further investigations are warranted to add MCLZ to the treatment protocol for various neuroinflammatory disorders.

## Conclusion

To sum up, the current study identifies for the first time the protective anti-neuroinflammatory effects of MCLZ against LPS-induced neuroinflammation in mice. LPS administration resulted in significant neuroinflammation in mice as was revealed by significant inflammatory histopathological changes and positive GFAP immunohistochemical staining accompanied by significant elevations of brain tissue contents of IL-1β, TNF-α, NF-κβ, AKT, ERK and JNK. MCLZ treatment protects against neuroinflammation via the amelioration of inflammatory histopathological changes as well as GFAP neuronal & astrocyte immunohistochemical staining. MCLZ treatment also down-regulates the AKT/NF-κB/ERK/JNK signaling pathway. Consequently, MCLZ is anticipated as a potential protective candidate for the addition to the treatment protocol of neuroinflammation, further investigation is required.

## Data Availability

All data will be available upon request.

## Abbreviations

MCLZ: Meclizine
LPS: Lipopolysaccharide
IL-1β: Interleukin − 1 beta
TNF-α: Tumor necrosis factor-alpha
NF-κβ: Nuclear factor kappa- beta
AKT: Protein kinase B
ERK: Extracellular signal-regulated kinase
JNK: c-Jun N-terminal Kinase
